# Multimodal MBC-ATT: cross-modality attentional fusion of EEG-fNIRS for cognitive state decoding

**DOI:** 10.3389/fnhum.2025.1660532

**Published:** 2025-09-24

**Authors:** Yu Li, Lei Zhu, Aiai Huang, Jianhai Zhang, Peng Yuan

**Affiliations:** ^1^HDU-ITMO Joint Institute, Hangzhou Dianzi University, Hangzhou, China; ^2^School of Automation, Hangzhou Dianzi University, Hangzhou, China; ^3^Provincial Key Laboratory of Soft Matter & Biomedical Materials, Wenzhou Institute of the University of Chinese Academy of Sciences (WIUCAS), Wenzhou, China; ^4^School of Computer Science, Hangzhou Dianzi University, Hangzhou, China; ^5^The Affiliated Wuxi People's Hospital of Nanjing Medical University, Wuxi, China; ^6^Wuxi Medical Center, Nanjing Medical University, Wuxi, China; ^7^Wuxi People's Hospital, Wuxi, China

**Keywords:** brain-computer interface, cognitive task, deep learning, multimodal signals, multimodal fusion

## Abstract

With the rapid development of brain-computer interface (BCI) technology, the effective integration of multimodal biological signals to improve classification accuracy has become a research hotspot. However, existing methods often fail to fully exploit cross-modality correlations in complex cognitive tasks. To address this, this paper proposes a Multi-Branch Convolutional Neural Network with Attention (MBC-ATT) for BCI based cognitive tasks classification. MBC-ATT employs independent branch structures to process electroencephalography (EEG) and functional near-infrared spectroscopy (fNIRS) signals separately, thereby leveraging the advantages of each modality. To further enhance the fusion of multimodal features, we introduce a cross-modal attention mechanism to discriminate features, strengthening the model's ability to focus on relevant signals and thereby improving classification accuracy. We conducted experiments on the n-back and WG datasets. The results demonstrate that the proposed model outperforms conventional approaches in classification performance, further validating the effectiveness of MBC-ATT in brain-computer interfaces. This study not only provides novel insights for multimodal BCI systems but also holds great potential for various applications.

## 1 Introduction

Brain-Computer Interface (BCI) represents a cutting-edge human-machine interaction paradigm that establishes direct neural pathways between the brain and external devices, enabling users to bypass traditional neuromuscular channels for device control (Sharma and Meena, 2024). Based on the mode of signal acquisition, BCI systems are generally categorized into invasive and non-invasive approaches. Compared to invasive methods, non-invasive techniques offer superior clinical applicability due to their enhanced safety and user tolerance. These approaches eliminate the risks associated with surgical implantation, making them suitable for long-term monitoring and mobile applications (Jafari et al., 2023). Moreover, they avoid the ethical concerns linked to intracranial implants, rendering them more appropriate for large-scale population studies and translational clinical research (Värbu et al., 2022). Accordingly, non-invasive acquisition methods were adopted in the present study.

Electroencephalography (EEG) and functional near-infrared spectroscopy (fNIRS) are two representative non-invasive brain signal acquisition techniques that have been widely adopted in BCI research due to their safety and ease of use (Liu et al., 2021). EEG offers millisecond-level temporal resolution, enabling real-time recording of cortical neural activity. This makes it particularly suitable for investigating high-level cognitive functions such as attention and memory. However, as EEG signals are collected via scalp electrodes, they are highly susceptible to artifacts from electromyographic activity, eye movements, and environmental noise (Li et al., 2022). Additionally, EEG exhibits considerable inter-subject variability, and the same subject's signal characteristics may change across sessions or experimental contexts. This variability hinders the cross-subject generalizability of EEG-based BCI systems and often necessitates a laborious calibration process for new users, which severely limits practical deployment (Wu et al., 2020). In contrast, fNIRS measures hemodynamic responses associated with neural activity by detecting changes in oxygenated and deoxygenated hemoglobin concentrations in the brain. It offers better spatial resolution and is more robust against motion artifacts, making it well-suited for experiments in more naturalistic settings (Chiarelli et al., 2017). However, fNIRS suffers from limited temporal resolution and is less capable of capturing fast neural dynamics.

To overcome this limitation, researchers have focused in recent years on developing multimodal fusion techniques. Among these, the combined application of EEG and fNIRS has garnered particular attention. The high temporal resolution of EEG complements the spatial localization capability of fNIRS, which indirectly reflects neural activity by detecting changes in cortical blood oxygen levels. Moreover, the strong resistance of fNIRS to artifacts such as eye movements effectively compensates for the inherent limitations of EEG (Lin et al., 2023). This fusion strategy not only deepens the multidimensional analysis of brain activity but also demonstrates significant value in areas such as motor imagery decoding, cognitive state assessment, and the diagnosis of neurological disorders.

In recent years, an increasing number of studies have attempted to enhance brain state recognition performance through multimodal fusion. For example, a method combining handcrafted features and traditional machine learning techniques was proposed (Cao et al., 2022) to classify multi-level brain load, but it heavily relies on complex preprocessing and feature extraction processes. To overcome the limitations of traditional approaches, some studies have begun to incorporate deep learning techniques. A novel recurrence plot (RP)-based time-distributed convolutional neural network and long short-term memory (CNN-LSTM) framework (Mughal et al., 2022) has been introduced for the integrated classification of EEG and fNIRS signals in hybrid BCI applications, demonstrating an effective approach for capturing spatiotemporal patterns across modalities. Additionally, short-time Fourier transform (STFT) has been employed to convert EEG signals into time-frequency images, which are subsequently integrated with the frequency-domain features of fNIRS using the Dense Convolutional Network (DenseNet) architecture, offering a complementary strategy for enhancing multimodal representation and classification performance in hybrid BCI systems (Bunterngchit et al., 2024). However, these methods largely depend on simple concatenation or stacking fusion strategies and fail to fully exploit the complementary and synergistic relationships between modalities.

In terms of fusion strategies, existing studies have explored different fusion timings and methods, which are generally categorized into early fusion–where multimodal features are combined at the input or low-level feature stage and late fusion, where decisions or high-level features from each modality are integrated at a later stage. For example, a comparison between early and late fusion approaches (Li et al., 2023) showed that early fusion, where multimodal features are combined before classification, can somewhat improve model performance. In contrast, a polynomial fusion method was proposed in Sun et al. (2020), which operates at a deeper semantic level and thus falls under the category of late fusion. Similarly, the FGANet model (Kwak et al., 2022) employs spatial mapping and attention mechanisms to extract high-level cross-modal features, serving as another example of a late fusion strategy that provides novel insights into improving representational capacity. However, current fusion methods still face several challenges: firstly, the ability to model complementary relationships between modalities is limited, lacking mechanisms for deeply exploring the dynamic dependencies between EEG and fNIRS (Bourguignon et al., 2022; Khan et al., 2021); secondly, most fusion methods rely on feature concatenation or static weighting, making it difficult to automatically focus on key modalities or brain region signals based on different task states (Nour et al., 2021; Chen et al., 2023).

To address these issues, this paper proposes a cross-modal attention fusion framework, Multimodal MBC-ATT. This framework is based on a late fusion strategy and incorporates a modality-guided attention mechanism aimed at selectively integrating information through the joint modeling of cross-modal features, thereby enhancing the decoding ability of cognitive states and overcoming the limitations of current methods in dynamic dependency modeling and task adaptability. The innovations of this study are primarily reflected in the following aspects:

- Cross-modal attention fusion framework (Multimodal MBC-ATT): by incorporating a modality-guided attention mechanism, the framework selectively integrates EEG and fNIRS signals, tackling the issue of inadequate modeling of complementary relationships between modalities in traditional methods.
- Dynamic dependency modeling: this approach overcomes the limitations of static fusion strategies by enabling the model to automatically concentrate on key modalities and brain region signals in accordance with task states, thereby enhancing task adaptability.
- Enhanced cross-modal synergy: compared to traditional feature concatenation or static weighting methods, this approach dynamically adjusts the contribution of each modality, enhancing the synergistic ability of fused signals and improving the decoding accuracy and robustness of the brain-computer interface.

## 2 Dataset and method

In this section, we describe the multimodal dataset that integrates EEG and fNIRS signals, outline the preprocessing steps necessary for effective signal representation, and finally present the MBC-ATT framework designed for cross-modal brain activity decoding.

### 2.1 Experimental dataset

This study used an open-access multimodal brain imaging dataset, which simultaneously recorded EEG and fNIRS signals, aiming to promote the development of neuroimaging analysis and BCI research (Shin et al., 2018). The dataset was collected from 26 healthy participants while performing three cognitive tasks, with the aim of providing high-quality multimodal signal data for BCI and neuroscience research. All participants were right-handed adults (nine males, 17 females) aged 17–33 years (*M* = 26.1, SD = 3.5). Each participant provided written informed consent and confirmed absence of neurological or psychiatric history through standardized screening questionnaires.

During the experiment, participants sat on a comfortable chair approximately 1.2 meters from a 24-inch LCD monitor, with their right index and middle fingers positioned on a numeric keypad for keypress responses. Three cognitive tasks were employed: the n-back task (Dataset A) to assess working memory load, the discrimination/selection response (DSR) task (Dataset B) to examine neural responses to target versus non-target stimuli, and the word generation (WG) task (Dataset C) to investigate brain activity related to language processing.

In this study, participants completed the n-back task (Dataset A) and the WG task (Dataset C) to investigate the multimodal neural signal characteristics under different cognitive task conditions.

**Figure 1:**
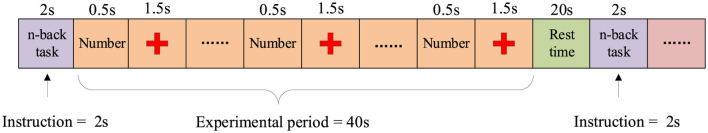
n-back task paradigm flowchart.

In the n-back task, participants made responses based on the task type: in the 0-back task, participants pressed the right index finger (target button) or the right middle finger (non-target button); in the 2-back and 3-back tasks, participants determined whether the currently displayed number matched the number shown 2 or 3 trials earlier and pressed the corresponding button. As shown in Figure 1, each task block consists of a 2-s instruction display, followed by a 40-s task period. During the task period, a random one-digit number is displayed every 2 s for 0.5 s, followed by a 1.5-s display of a fixed cross. After the task period, participants enter a 20-s rest period, during which they focus on a fixed cross displayed on the screen. Each participant performed a total of 180 trials (20 trials × 3 series × 3 sessions) to ensure the adequacy and reliability of the data.

**Figure 2:**
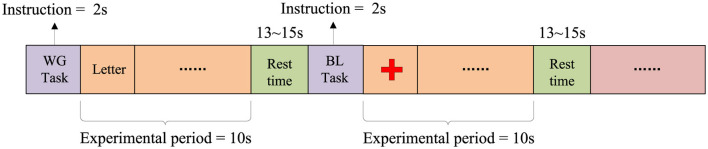
WG task paradigm flowchart.

The WG task is a spontaneous word generation task in which participants are required to think of and silently generate words starting with a specific letter within a limited time, aiming to investigate the neural activity characteristics of brain regions related to language. The experiment consists of 3 sessions, each containing 10 WG tasks and 10 Baseline (BL) tasks. Participants perform a total of 30 WG tasks and 30 BL tasks. As shown in Figure 2, each trial consists of a 2-s task prompt, a 10-s task execution period, and a 13–15 s rest period. During the WG task period, a random letter is displayed on the screen, and participants are required to quickly generate and silently list as many words as possible starting with that letter within 10 s, while avoiding repetition. In the BL task period, participants are instructed to focus on a fixed point at the center of the screen to maintain a low cognitive load, serving as a control condition for the WG task.

The EEG-fNIRS multimodal dataset used in this study underwent basic preprocessing prior to its release to ensure data quality. The EEG data were sampled at 1,000 Hz, consisting of 30 EEG channels and 2 Electrooculography (EOG) channels, and were downsampled to 200 Hz during the data processing stage. A 1–40 Hz bandpass filter was applied to remove low-frequency drift and high-frequency noise, as this frequency range preserves task-related neural oscillations (theta, alpha, beta, and low gamma) while suppressing irrelevant artifacts. Eye movement artifacts were removed using the EEGLAB toolbox (Martínez-Cancino et al., 2021). The fNIRS data consist of 36 channels with a sampling rate of 10.4 Hz. The raw optical intensity measurements were converted to changes in concentrations of oxyhemoglobin (HbO) and deoxyhemoglobin (HbR), and the sampling rate was downsampled to 10 Hz. Additionally, basic artifact removal and noise filtering were applied during data acquisition.

To meet the requirements of this experiment, further processing was performed on the data. First, the EEG and fNIRS signals were synchronized and segmented based on the task time markers. The 2 s before the task start and the rest period after the task were discarded, retaining only the valid data following the task onset. Each task data was segmented into multiple time windows using a sliding window approach (with a window length of 5 s and a step size of 1 s), which was chosen to ensure that each segment contains sufficient task-related neural activity while increasing the number of samples and maintaining temporal continuity for more robust analysis.

### 2.2 Methods

**Figure 3:**
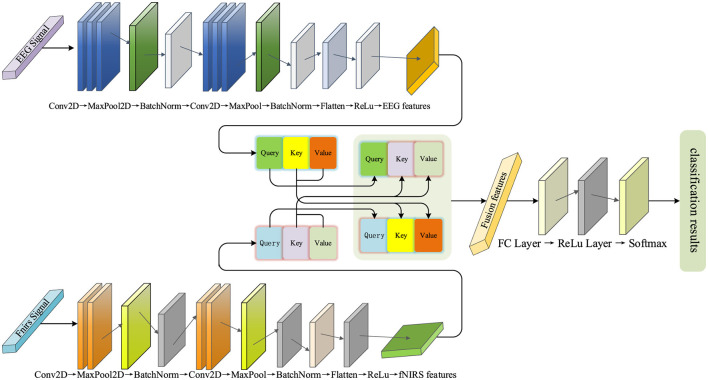
MBC-ATT network architecture.

The paper proposes a decoding framework that integrates a multi-branch convolutional neural network with a cross-modal attention mechanism (MBC-ATT), aimed at efficient joint modeling of EEG and fNIRS signals. The model independently extracts temporal and spatial features of EEG and fNIRS signals through separate branches and introduces a modality-guided attention mechanism to achieve dynamic fusion and selective enhancement of cross-modal features, thereby improving decoding performance for complex cognitive tasks. The network architecture is illustrated in Figure 3.

The model employs a dual-branch architecture, with the upper branch corresponding to the EEG feature extraction module and the lower branch dedicated to the fNIRS feature extraction module. The features extracted from both modalities are subsequently fed into a cross-modal attention fusion mechanism to accomplish the final classification task.

#### 2.2.1 EEG feature extraction network

This branch uses a convolutional neural network (CNN) as the core architecture for the EEG feature extraction branch. The branch aims to automatically extract meaningful features from raw EEG signals using deep learning methods, thereby enhancing the performance of multimodal fusion.

This branch consists of multiple convolutional layers, pooling layers, and fully connected layers. In the initial stage, three convolutional layers with kernel sizes of (7, 1) are used to gradually extract local features from the EEG signals, with the ReLU activation function introducing non-linearity to enhance the feature representation capability. This kernel configuration is primarily based on the temporal characteristics of EEG signals, where the (7,1) kernel slides along the time dimension to capture short-term local temporal dependencies while maintaining the independence across channels. This design was inspired by Yu et al. (2025), which demonstrated that short temporal receptive fields are effective for EEG decoding; in our work, the (7,1) kernel was further adapted to our data characteristics (sampling rate and channel configuration) to balance temporal locality with computational efficiency. Subsequently, a max pooling layer is applied to downsample the feature map, reducing its spatial dimensions while preserving key information. This operation effectively reduces computational complexity and enhances the robustness of the features. Additionally, batch normalization (Ogundokun et al., 2022) is applied to standardize the features, accelerating the model training and improving its stability. In the deeper layers of the network, three two-dimensional convolutional layers with kernel sizes of (4 × 4) are used to extract global EEG features, a configuration designed to capture spatial correlations across multiple channels, which helps identify more complex inter-channel patterns and reflect the spatial distribution of brain activity. Max pooling is again applied to further downsample the feature map for increased feature abstraction, while batch normalization continues to optimize the training process. Finally, the feature maps are flattened and passed through two fully connected layers to extract high-level features and perform feature mapping, thereby enhancing discriminability and providing optimized feature representations for multimodal fusion in classification tasks.

#### 2.2.2 fNIRS feature extraction network

Unlike EEG signals, fNIRS signals reflect changes in blood oxygen concentration, with feature extraction focusing more on local fluctuations and temporal changes. Despite their differences in physiological characteristics, both utilize CNN as the core architecture. This design ensures consistency in multimodal data processing, while also reducing the complexity of module design, thereby facilitating subsequent multimodal fusion.

The initial stage of the network consists of two convolutional layers with kernel sizes of (4, 1), designed to extract local spatial features of the fNIRS signals, particularly modeling the temporal dynamics of blood oxygen concentration. Subsequently, the pooling layer downsamples the feature map, reducing its spatial dimensions, while preserving key features and lowering computational complexity. In the subsequent layers, the network further extracts complex spatial features through two convolutional layers with kernel sizes of (2, 2), identifying blood oxygen concentration variation patterns between different regions. These convolutional layers progressively expand the receptive field, capturing broader spatial information. Finally, the fully connected layers merge and transform the extracted spatiotemporal features of blood oxygen concentration, generating high-level features that serve as input for subsequent classification and multimodal fusion. This design enables the fNIRS feature extraction branch to efficiently capture the spatial distribution and temporal dynamics of oxygen hemodynamics and map them to a feature space suitable for multimodal tasks.

#### 2.2.3 Multimodal feature fusion network

In this study, to fully leverage the complementarity of the two modalities, we employed an attention mechanism (Vaswani et al., 2017) to dynamically capture the dependencies between them.

First, the input features of EEG and fNIRS are mapped onto a unified hidden space using independent linear transformation layers. This step ensures that both modalities, which may have different original dimensions or feature distributions, are projected into a common representational space, facilitating effective interaction and comparison. Subsequently, the features of the EEG and fNIRS modalities are passed through independent linear transformation layers to generate their corresponding Query, Key, and Value representations, which are used to assess both intra-modal and inter-modal dependencies. These additional transformations allow the model to learn optimal representations tailored for attention computation, enabling it to focus on the most relevant parts of the input. To enhance the interaction between modalities, the model employs a cross-modal attention mechanism for fusion. Specifically, the Query from the EEG modality interacts with the Key and Value from the fNIRS modality, and similarly, the fNIRS modality interacts with the EEG modality. This cross-modal fusion allows each modality to dynamically reference the features of the other, thereby fully leveraging the complementary nature of the high temporal resolution of EEG and the spatial resolution of fNIRS. In addition, the model employs a 4-head attention mechanism, where the Query, Key, and Value are divided into four independent subspaces. Each head captures the inter-modal dependencies from a different perspective. This multi-head design further enhances the expressive power of the attention mechanism, enabling it to model the complex the complex relationships between EEG and fNIRS more comprehensively.

## 3 Experiments and results

In the experiments, this study employs the n-back task and the WG task to validate the effectiveness of the proposed MBC-ATT method across different cognitive tasks. The experimental procedure strictly follows standardized protocols for data splitting, feature extraction, and model training to ensure the reproducibility of the results.

All experiments were conducted on a system equipped with an Intel Core processor and an NVIDIA GeForce RTX 4060 Laptop GPU. The software environment included Python 3.10 and PyTorch 2.1 with CUDA 11.8 support, all implemented within an Anaconda-managed virtual environment. For the n-back experiment, the model was trained for 60 epochs using the Adam optimizer with an initial learning rate of 1e-3; for the WG experiment, training lasted 70 epochs with the same optimizer and learning rate. Other training hyperparameters were kept consistent across both experiments.

### 3.1 Experimental plan

This study adopts a within-subject partitioning strategy to evaluate the applicability of the model at the individual level. By performing independent training and testing on the dataset of each subject, confounding effects arising from inter-individual variability are effectively mitigated. This approach not only enhances recognition accuracy but also ensures robust adaptation to subject-specific physiological signal characteristics.

For each subject, the dataset is randomly partitioned into training (80%) and testing (20%) subsets, with the former dedicated to model development and the latter reserved for final performance assessment. To ensure robustness against partitioning randomness, the training subset undergoes five-fold cross-validation. This strategy not only mitigates overfitting but also enhances the model's generalizability to unseen data.

To further ensure the robustness and generalizability of the model, we additionally conducted a complementary evaluation using five-fold cross-validation on the entire dataset. This cross-validation procedure mitigates overfitting and reduces potential bias caused by data partitioning, providing a more comprehensive assessment of the model's performance.

### 3.2 Evaluation metrics

To provide a comprehensive evaluation of the classification model's performance, this study utilizes four key metrics: Accuracy, Precision, Recall, and the F1-score.

Accuracy serves as a cornerstone evaluation metric in deep learning-based classification tasks, formally defined as the ratio between correctly predicted instances and the total number of test samples. The computation follows the standard formulation:


(1) $$\begin{array}{l} \text{Accuracy}=\frac{TP+TN}{TP+TN+FP+FN} \end{array}$$

In the classification framework, True Positives (TP) correspond to the number of positive instances correctly identified by the model, True Negatives (TN) represent correctly classified negative cases, False Positives (FP) indicate negative samples erroneously predicted as positive, and False Negatives (FN) signify positive samples inaccurately classified as negative.

Precision quantifies the model's predictive reliability for the positive class, representing the proportion of true positives among all positive predictions. The metric is formally defined as:


(2) $$\begin{array}{l} \text{Precision}=\frac{TP}{TP+FP} \end{array}$$

Elevated precision demonstrates the model's enhanced accuracy in positive-class identification, characterized by reduced false positive predictions. This metric proves especially critical in applications where misclassification entails significant consequences.

Recall quantifies the model's sensitivity in detecting positive-class instances, defined as the ratio of true positives to all actual positives in the population. The formal computation is expressed as:


(3) $$\begin{array}{l} \text{Recall}=\frac{TP}{TP+FN} \end{array}$$

An elevated recall rate demonstrates the model's enhanced detection capability for positive instances, albeit with a potential compromise in specificity through increased false positives. This performance metric assumes critical importance in high-stakes applications where false negatives may incur substantial costs, such as medical diagnosis, security surveillance, or fault detection systems.

The F1-score represents the harmonic mean of precision and recall, providing a balanced metric that reconciles the trade-off between these two competing objectives. Formally, it is computed as:


(4) $$\begin{array}{l} \text{F1-score}=\frac{2\times \text{Precision}\times \text{Recall}}{\text{Precision}+\text{Recall}} \end{array}$$

Under class-imbalanced conditions, the F1-score serves as a robust composite metric for evaluating overall model performance, mitigating potential assessment bias induced by over-reliance on individual metrics such as precision or recall in isolation.

Furthermore, to enable intuitive cross-class performance analysis, this investigation incorporates confusion matrix visualization (Lei et al., 2016). This diagnostic tool explicitly maps the correspondence between ground-truth and predicted labels, uncovering category-specific classification biases that inform targeted model refinement.

### 3.3 Experimental results

#### 3.3.1 n-back dataset experimental results

In the n-back experiment, we followed the established experimental plan for data partitioning and model training, and evaluated the model performance on the test set.

We computed multiple evaluation metrics for each subject, as shown in Table 1. The results indicate that the proposed method demonstrates excellent classification performance at the individual level. Although there are differences in classification performance across different subjects, the method is able to adapt to inter-individual variations in neural signals and maintains a high classification accuracy in most subjects, demonstrating good generalization ability.

**Table 1:** Classification performance metrics of each subject in the n-back task.

**Subject 01–13**				**Subject 15–26**			
**Accuracy**	**Precision**	**Recall**	**F1 score**	**Accuracy**	**Precision**	**Recall**	**F1 score**
0.9697	0.9697	0.9697	0.9683	0.9999	0.9999	0.9999	0.9999
0.9999	0.9999	0.9999	0.9999	0.9091	0.9139	0.8956	0.9015
0.9697	0.9762	0.9667	0.9701	0.9999	0.9999	0.9999	0.9999
0.9999	0.9999	0.9999	0.9999	0.9999	0.9999	0.9999	0.9999
0.9999	0.9999	0.9999	0.9999	0.9697	0.9697	0.9697	0.9683
0.9999	0.9999	0.9999	0.9999	0.9999	0.9999	0.9999	0.9999
0.9999	0.9999	0.9999	0.9999	0.9999	0.9999	0.9999	0.9999
0.9999	0.9999	0.9999	0.9999	0.9999	0.9999	0.9999	0.9999
0.9999	0.9999	0.9999	0.9999	0.9697	0.9744	0.9792	0.9759
0.9999	0.9999	0.9999	0.9999	0.9091	0.9172	0.9167	0.9122
0.9999	0.9999	0.9999	0.9999	0.9999	0.9999	0.9999	0.9999
0.9999	0.9999	0.9999	0.9999	0.9999	0.9999	0.9999	0.9999
0.8182	0.8222	0.8056	0.8042	0.9999	0.9999	0.9999	0.9999

To further analyze the overall performance of the model, we calculated the average results of all subjects. The classification accuracy reached 98.13%, precision was 98.24%, recall was 98.10%, and the F1 score was 98.11%. This result validates the effectiveness and robustness of the model in a within-subject partition scenario. Furthermore, the high consistency observed across different subjects further demonstrates the model's robustness to individual neural signal variations, as it can stably extract common features and perform classification tasks.

**Figure 4:**
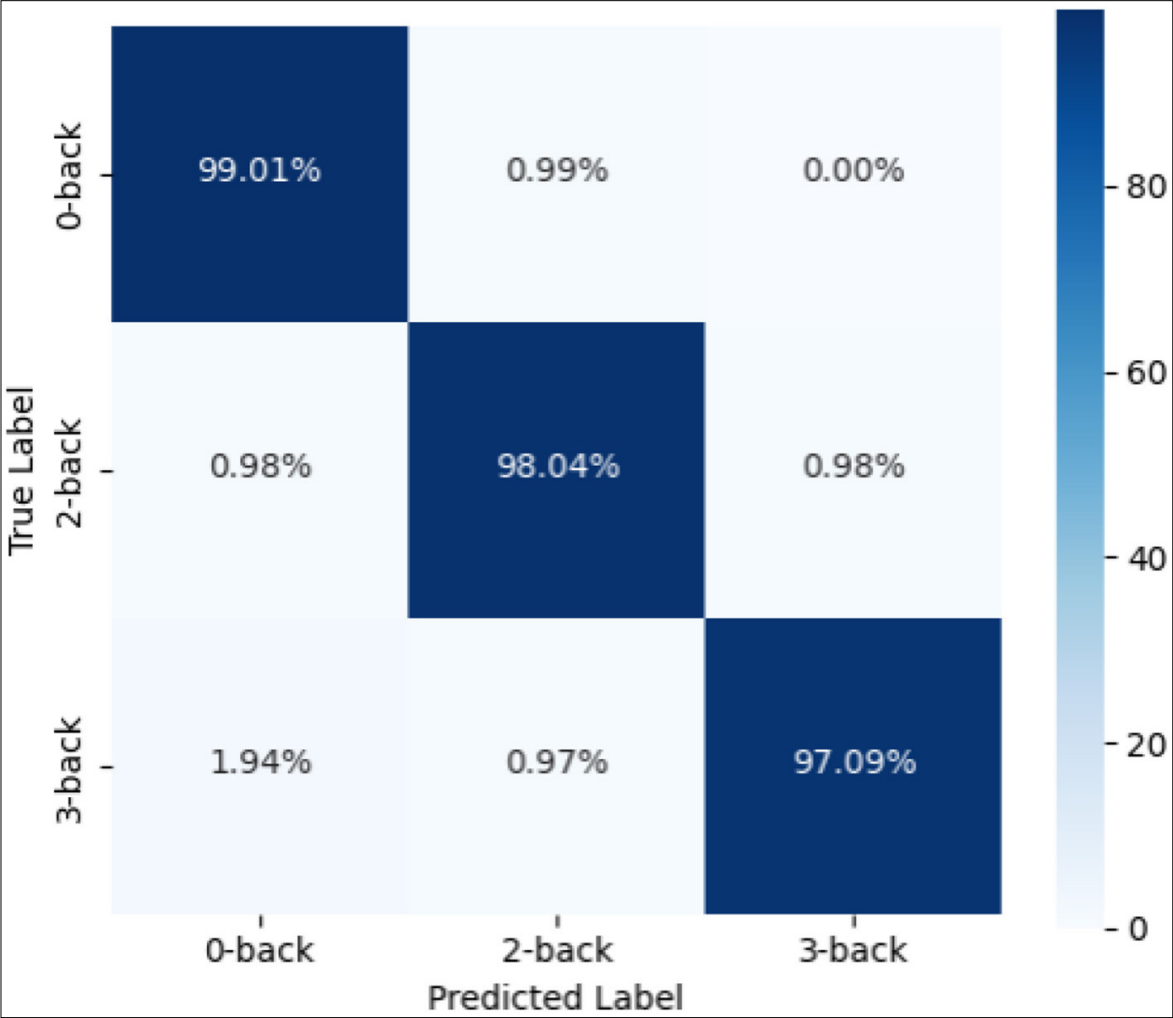
Confusion matrix of n-back experiment results.

In addition, we plotted the confusion matrix (as shown in Figure 4) to further analyze the classification performance of the model. From the figure, it can be seen that the model's prediction results are well-balanced across categories with a high accuracy, indicating that the proposed method has strong discriminative ability across different task categories.

#### 3.3.2 WG dataset experimental results

Similar to the n-back dataset, we followed the same experimental procedure for data partitioning, model training, and performance evaluation on the test set. As shown in Table 2, the proposed method achieves high classification accuracy for each subject. The average classification accuracy reaches 98.61%, the precision is 99.79%, the recall is 97.44%, and the F1-score is 98.58%.

**Table 2:** Classification performance metrics of each subject in the WG task.

**Subject 01–13**				**Subject 15–26**			
**Accuracy**	**Precision**	**Recall**	**F1 score**	**Accuracy**	**Precision**	**Recall**	**F1 score**
0.9583	0.9999	0.9167	0.9565	0.9999	0.9999	0.9999	0.9999
0.9861	0.9999	0.9722	0.9859	0.9722	0.9999	0.9444	0.9714
0.9999	0.9999	0.9999	0.9999	0.9999	0.9999	0.9999	0.9999
0.9722	0.9722	0.9722	0.9722	0.9722	0.9999	0.9722	0.9859
0.9444	0.9999	0.8889	0.9412	0.9861	0.9999	0.9722	0.9859
0.9861	0.9730	0.9999	0.9863	0.9722	0.9999	0.9444	0.9714
0.9999	0.9999	0.9999	0.9999	0.9999	0.9999	0.9999	0.9999
0.9722	0.9999	0.9444	0.9714	0.9861	0.9999	0.9722	0.9859
0.9999	0.9999	0.9999	0.9999	0.9722	0.9999	0.9444	0.9714
0.9861	0.9999	0.9722	0.9859	0.9999	0.9999	0.9999	0.9999
0.9861	0.9999	0.9722	0.9859	0.9722	0.9999	0.9722	0.9859
0.9722	0.9999	0.9444	0.9714	0.9999	0.9999	0.9999	0.9999
0.9861	0.9999	0.9722	0.9859	0.9861	0.9999	0.9722	0.9859

**Figure 5:**
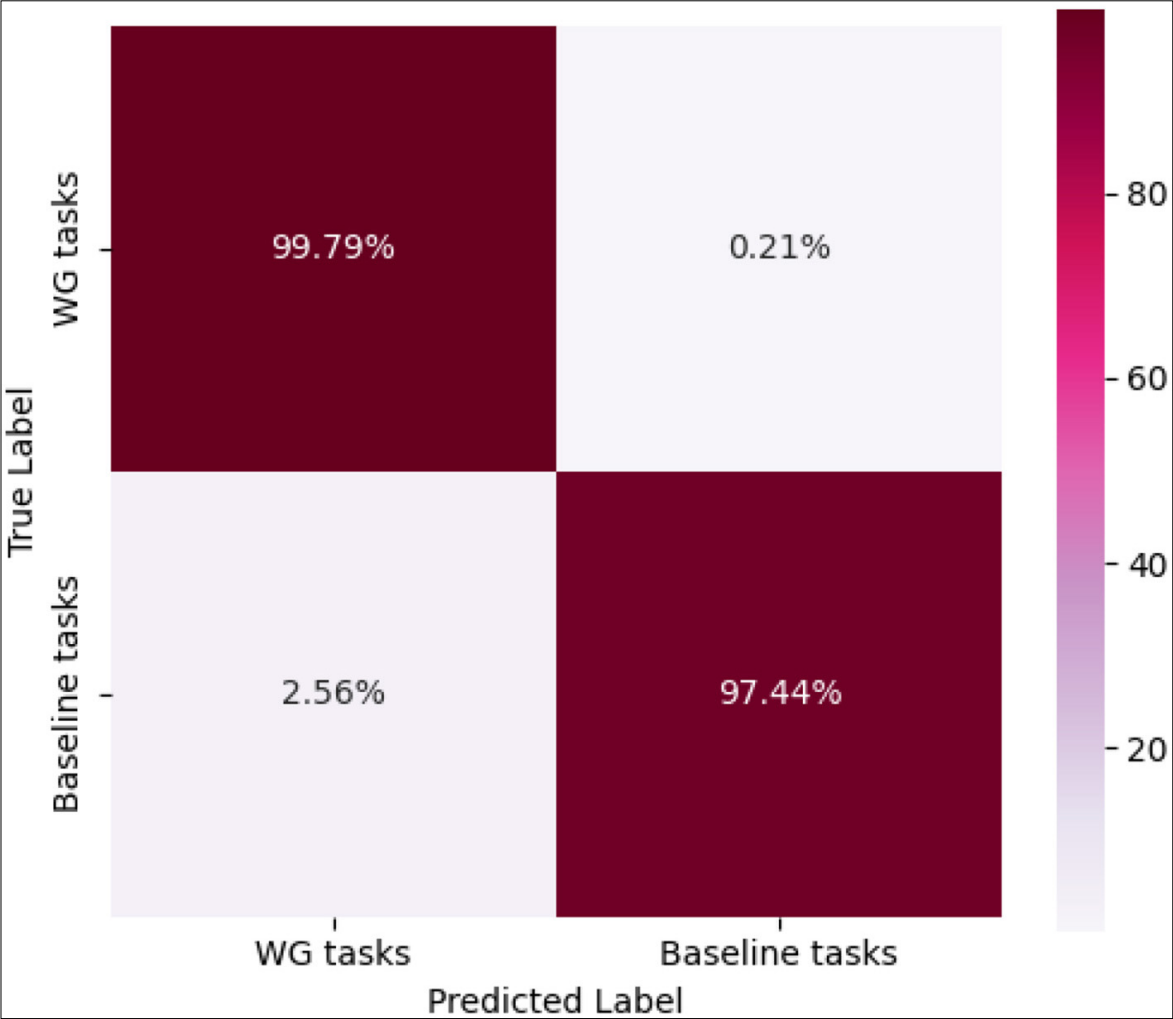
Confusion matrix of WG experiment results.

The model's classification performance was further analyzed using the confusion matrix (as shown in Figure 5). The results indicate that the recognition outcomes are well-balanced across different categories, with diagonal elements significantly larger than non-diagonal elements. This demonstrates a high correct classification rate and a low misclassification rate, highlighting the model's robustness.

### 3.4 Model evaluation with cross-validation and results

To enhance the robustness and generalizability of the model evaluation, the original 80:20 train-test split strategy was replaced with a five-fold cross-validation approach. This adjustment aims to better address potential overfitting issues and provide a more comprehensive assessment of the model's performance. Specifically, the dataset was randomly divided into five equally sized subsets. In each fold, one subset was used as the test set while the remaining four were used for training. This process was repeated five times, ensuring that each sample was tested exactly once. The final performance metrics, including accuracy, precision, recall, and F1-score, were averaged across all folds to obtain a more reliable and stable evaluation.

Table 3 presents the mean accuracy, precision, recall, and F1-score of the model evaluated via five-fold cross-validation on the n-back and WG datasets. The results demonstrate that the model achieves consistently high and stable performance across both datasets, with accuracies of approximately 97.53 and 96.68%, and F1-scores of 97.54 and 96.34%, respectively. The close alignment between accuracy and F1-score indicates the model's balanced capability across classes, ensuring both precise classification and overall performance stability.

**Table 3:** Performance of the model on n-back and WG datasets under five-fold cross-validation.

**Dataset**	**Metric**	**Fold 1**	**Fold 2**	**Fold 3**	**Fold 4**	**Fold 5**	**Average**
n-back	Accuracy	0.9739	0.9775	0.9786	0.9739	0.9727	**0.9753**
	Precision	0.9750	0.9776	0.9790	0.9740	0.9723	**0.9756**
	Recall	0.9732	0.9772	0.9785	0.9741	0.9734	**0.9753**
	F1-score	0.9739	0.9774	0.9787	0.9740	0.9728	**0.9754**
WG	Accuracy	0.9602	0.9639	0.9762	0.9709	0.9628	**0.9668**
	Precision	0.9550	0.9605	0.9750	0.9698	0.9600	**0.9640**
	Recall	0.9520	0.9585	0.9748	0.9696	0.9584	**0.9627**
	F1-score	0.9535	0.9595	0.9749	0.9697	0.9592	**0.9634**

The bold font represents the average value of the previous five fold cross validation.

### 3.5 Effectiveness of the cross-modal attention mechanism

To evaluate the contribution of the multimodal fusion module to overall model performance, this study designed and conducted an ablation experiment. Specifically, in the ablated model, the cross-modal attention mechanism originally proposed in the model was removed, and the Query-Key-Value structure used to model the dependency between EEG and fNIRS modalities was no longer employed. Accordingly, the high-level features extracted from the two modalities were directly concatenated and fed into the classifier for decision-making. This modification retained the unimodal feature extraction structures but omitted the explicit feature interaction mechanism between modalities, serving as a baseline for comparison against the proposed multimodal fusion strategy in the controlled experiments.

On the nback task, the classification accuracy of the original fusion model reached 98.13%, whereas it dropped to 91.58% after removing the fusion module. On the WG dataset, the accuracy decreased from 98.61 to 96.54%. These results demonstrate that the proposed fusion module effectively enhances model performance across both datasets. Notably, the accuracy improvement of 6.55 percentage points on the nback dataset highlights a more pronounced advantage of the fusion mechanism in capturing complementary information between modalities.

In summary, the designed fusion mechanism plays a critical role in leveraging the complementary information between EEG and fNIRS modalities and enhancing feature representation capabilities, thereby effectively improving the model's discriminative power and generalization performance.

### 3.6 Statistical verification of results

**Figure 6:**
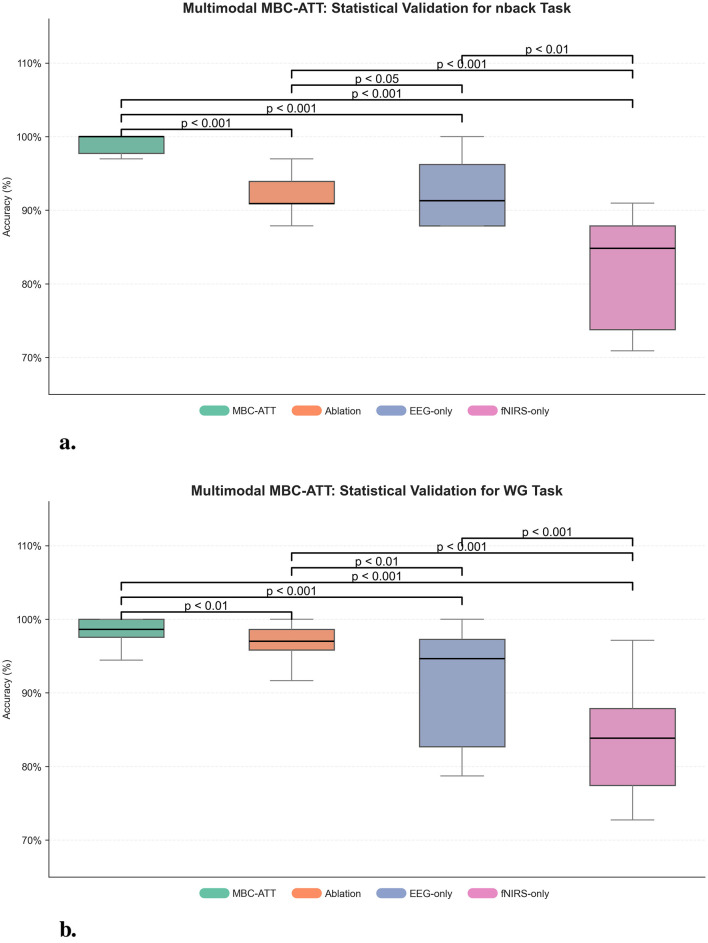
Multimodal MBC-ATT: statistical validation. **(a)** Statistical validation for n-back task. **(b)** Statistical validation for WG task.

To quantitatively assess the significance of the performance improvements introduced by MBC-ATT, paired t-tests were conducted comparing MBC-ATT with three baseline models: the ablation model, the EEG-only model, and the fNIRS-only model. The results, presented in Figure 6, indicate that MBC-ATT consistently outperforms all baseline models. The obtained p-values are statistically significant (*p* < 0.05), confirming that the multimodal fusion contributes substantially to improvements in classification accuracy.

On the nback and WG tasks, MBC-ATT effectively captures complementary features from EEG and fNIRS, thereby enhancing feature representation. The paired t-test results further confirm that these improvements are statistically significant, indicating that the high accuracies are robust and reliable.

### 3.7 Comparative analysis of methods

To comprehensively evaluate the performance of the proposed method, we conducted a comparative analysis with methods from relevant literature for both the n-back and WG tasks. Given the differences in cognitive load and signal characteristics between these two tasks, the selected comparison methods also vary accordingly. The following sections provide a detailed discussion of the comparisons for the n-back and WG tasks, respectively.

#### 3.7.1 Performance comparison of the n-back task

To comprehensively evaluate the performance of the proposed method, we compared it with four existing approaches, including Support Vector Machine (SVM) (Wu et al., 2020), Deep Neural Network (DNN) (Vaswani et al., 2017), a time-distributed CNN-LSTM method based on recurrence plots (CNN-LSTM) (Chiarelli et al., 2017), and a multimodal DenseNet fusion model based on Short-Time Fourier Transform (STFT-MDNF) (Lin et al., 2023).

As shown in Table 4, this study systematically compares the classification performance of five methods in the n-back task. Compared to SVM (83.00%), DNN (87.00%), CNN-LSTM (88.41%), and STFT-MDNF (95.10%), the MBC-ATT method (98.13%) achieves accuracy improvements of 15.13%, 11.13%, 9.72%, and 3.03%, respectively, significantly outperforming existing methods. These results strongly demonstrate the superior performance of the MBC-ATT method in cognitive load recognition and classification for the n-back task.

**Table 4:** Performance comparison of different methods on the n-back task.

**Method**	**Accuracy (%)**
SVM	83.00%
DNN	87.00%
CNN-LSTM	88.41%
STFT-MDNF	95.10%
**MBC-ATT**	**98.13%**

The bold font represents the experimental results of the MBC-ATT method proposed in this manuscript.

#### 3.7.2 Performance comparison of the WG task

This study focuses on the characteristics of the WG task and compares four methods: Support Vector Machine (SVM) (Wu et al., 2020), Deep Neural Network (DNN) (Vaswani et al., 2017), EEG-fNIRS Convolutional Network (EF-Net) (Arif et al., 2024), and a Short-Time Fourier Transform-based Multimodal DenseNet Fusion Model (STFT-MDNF) (Lin et al., 2023). The primary focus is to evaluate the performance advantages of the proposed MBC-ATT model in the WG task.

As shown in Table 5, the proposed MBC-ATT method demonstrates superior performance in the WG task, achieving a classification accuracy of 98.61%. The performance comparison indicates that MBC-ATT improves accuracy by 24.62 percentage points over the traditional SVM method (73.99%), 6.61 percentage points over the deep neural network (DNN) (92.00%), 5.51 percentage points over STFT-MDNF (93.10%), and 2.32 percentage points over EF-Net (96.29%). These results strongly confirm the superior effectiveness of the MBC-ATT method in the WG task.

**Table 5:** Performance comparison of different methods on the WG task.

**Method**	**Accuracy (%)**
SVM	73.99%
DNN	92.00%
STFT-MDNF	93.10%
EF-Net	96.29%
**MBC-ATT**	**98.61%**

The bold font represents the experimental results of the MBC-ATT method proposed in this manuscript.

## 4 Discussion

**Figure 7:**
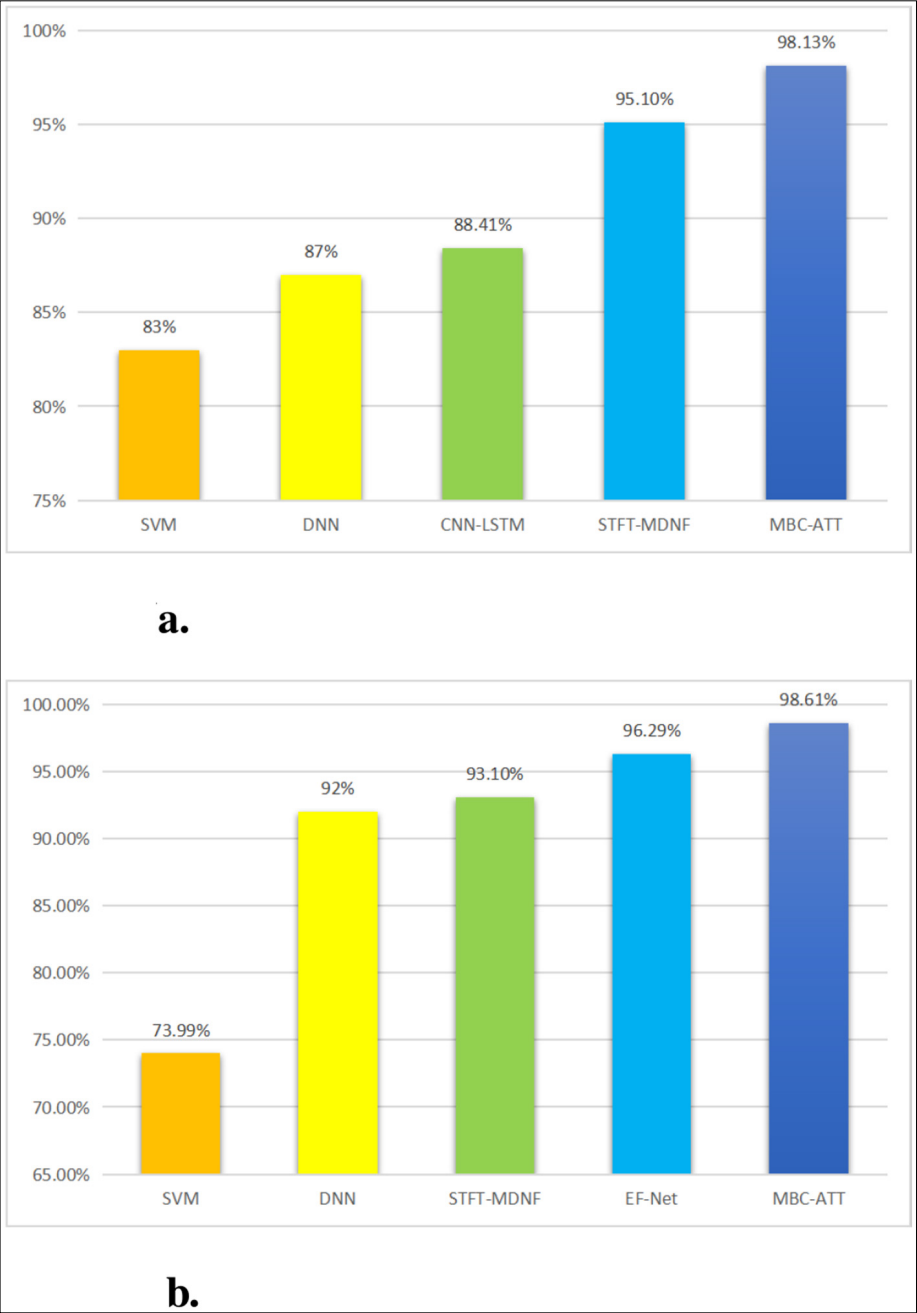
Comparison of experimental results. **(a)** Classification performance on the n-back task. **(b)** Classification performance on the WG task.

The results of this study indicate that the Multimodal MBC-ATT framework significantly enhances classification accuracy in both the n-back and WG tasks, effectively demonstrating the feasibility and advantages of its cross-modal attention mechanism for EEG and fNIRS signal fusion. The framework achieved classification accuracies of 98.13 and 98.61% in the two tasks, respectively, outperforming existing fusion methods (as shown in Figure 7).

The superior performance achieved in this study can be primarily attributed to the proposed framework's innovative cross-modal attention mechanism. This mechanism leverages the Query-Key-Value interaction strategy across modalities to adaptively balance the contributions of EEG signals, which are characterized by high temporal resolution, and fNIRS signals, which provide high spatial resolution. In doing so, it enables precise feature-level alignment and effective complementary fusion. The mechanism effectively mitigates the inherent discrepancies between EEG and fNIRS in terms of both temporal dynamics and physiological representations. Specifically, EEG reflects electrophysiological activity on a millisecond scale, while fNIRS captures hemodynamic responses on a second scale. Additionally, EEG represents neural electrical activity, while fNIRS reflects blood oxygen metabolism. By preserving the unique information of each modality, this approach significantly enhances the representation of task-relevant features, thereby offering a novel and effective fusion strategy to optimize the performance of multimodal brain-computer interface systems.

Compared with existing multimodal fusion approaches, the proposed MBC-ATT model demonstrates superior performance. In contrast to early fusion strategies, which typically concatenate features from different modalities and are prone to mutual interference and loss of modality-specific information, MBC-ATT employs a branch-specific feature extraction architecture that effectively preserves the unique characteristics of each modality. Moreover, unlike late fusion methods that often perform simple integration at the decision level and thus overlook cross-modal feature interactions, MBC-ATT incorporates an attention mechanism to enable dynamic fusion at the feature level. This design not only facilitates more effective inter-modal information exchange but also aligns well with the physiological basis of neurovascular coupling.

The proposed approach demonstrates significant application potential across various domains. Achieving a classification accuracy exceeding 98%, the model provides a robust foundation for the development of real-time BCI systems, particularly in scenarios such as cognitive workload monitoring and neurofeedback training. The attention weight distributions offer a novel methodology for quantifying functional connectivity strength across brain regions under different task states, thereby facilitating deeper investigations into multimodal brain network dynamics.

Despite these promising results, several limitations remain to be addressed. The current model requires a high degree of temporal synchronization between modalities, highlighting the need for future research into fusion strategies that can accommodate asynchronous signals. Additionally, the model's generalization ability on small-sample patient datasets has yet to be thoroughly validated and may benefit from the integration of techniques such as transfer learning. Furthermore, the relatively high computational cost poses challenges for real-time deployment. To address this, future work will explore lightweight solutions, including knowledge distillation, to enhance system efficiency. These advancements are expected to strengthen the practical applicability of the proposed method in real-world BCI applications.

## 5 Conclusion

This study introduces MBC-ATT for cognitive state classification utilizing EEG-fNIRS multimodal data. Extensive experimental evaluations demonstrate that MBC-ATT consistently achieves superior classification performance in both n-back and WG tasks, compared to traditional machine learning models and deep learning models.

The proposed MBC-ATT framework employs a multi-branch convolutional architecture to effectively extract spatiotemporal features from EEG and fNIRS signals. An integrated attention mechanism further enhances feature fusion by selectively emphasizing salient neural patterns. This design not only improves the model's discriminative capability for different cognitive states but also strengthens its generalization performance across subjects.

The experimental results substantiate the effectiveness of MBC-ATT in cognitive load recognition and spontaneous language generation tasks. Future research will focus on further optimizing the model architecture to enhance adaptability to individual variability, as well as exploring its potential applications in a wider range of cognitive paradigms and real-time BCI systems.

## Data Availability

The original contributions presented in the study are included in the article/supplementary material, further inquiries can be directed to the corresponding author.
